# Concurrent or Sequential Chemoradiotherapy after 3-4 Cycles Induction Chemotherapy for LS-SCLC with Bulky Tumor

**DOI:** 10.7150/jca.41136

**Published:** 2020-06-16

**Authors:** Jingjing Zhao, Wencheng Zhang, Puchun Er, Xi Chen, Yong Guan, Dong Qian, Jun Wang, Zhiyong Yuan, Lujun Zhao, Ping Wang, Qingsong Pang

**Affiliations:** Department of Radiation Oncology, Tianjin Medical University Cancer Institute and Hospital, National Clinical Research Center for Cancer, Key Laboratory of Cancer Prevention and Therapy, Tianjin, Tianjin's Clinical Research Center for Cancer, Tianjin 300060, China.

**Keywords:** chemotherapy, limited-stage, oral etoposide, small cell lung cancer, thoracic radiotherapy

## Abstract

The current study was to compare the efficacy and safety between concurrent and sequential chemoradiotherapy after 3-4 cycles of induction chemotherapy for limited-stage small-cell lung cancer (LS-SCLC) with bulky tumor. From July 2012 to September 2015, a total of 68 patients with stage IIIA and IIIB SCLC who had completed 3-4 cycles of etoposide plus cisplatin/carboplatin and achieved clinical complete response (cCR) or clinical partial response (cPR) were randomized into the two groups equally. The concurrent group received radiotherapy combined with oral etoposide and cisplatin and the sequential group received sequential chemoradiotherapy. Thoracic radiotherapy was performed using intensity-modulated radiation therapy (IMRT) with 95% PTV 60Gy/30 times. After completing chemoradiotherapy, patients received prophylactic cranial irradiation. The primary endpoint was progression-free survival (PFS), and secondary endpoints included overall survival (OS) and toxicity. The median follow-up time was 63.3 months (95% confidence interval [CI], 50.8-75.8). Better PFS and OS were observed in concurrent group (median PFS, 26.0 months [95% CI, 9.0-43.0] versus 13.1 months [95%CI, 9.7-16.6], p=0.023; median OS, 35.0 months [95% CI, 25.4-44.6] versus 22.0 months [95% CI, 17.0-27.1], p=0.015). There was no significant difference in the incidence of radiation esophagitis and radiation pneumonitis between the two groups (p=0.795, p=0.525). This study demonstrated that after the completion of 3-4 cycles of chemotherapy with a remission, concurrent chemoradiotherapy with oral etoposide and cisplatin improved survival compared with sequential chemoradiotherapy in LS-SCLC with bulky tumor. ClinicalTrials.gov Identifier: NCT01745445.

## Introduction

The concurrent thoracic radiotherapy (TRT) combined with chemotherapy has been proved to improve overall survival (OS) and considered to be the standard treatment for limited-stage small-cell lung cancer (LS-SCLC) 1-3. In the regimen of platinum chemotherapy concurrent with TRT, a significant higher 5-year survival was observed when chest radiotherapy was started within 30 days after the start of chemotherapy 4-7. Other studies failed to show a survival advantage of early TRT with chemotherapy for LS-SCLC 8, 9. In a Korean study of LS-SCLC, TRT starting in the third cycle of chemotherapy was not inferior to early TRT and had a more favorable profile with regard to neutropenic fever 10. However, patients with LS-SCLC always had bulky tumors that would require large radiation target volume with concomitant increase in acute or chronic toxicity. Chemoradiotherapy might be interrupted because of intolerance in most patients, thus therapeutic effect was affected. Compared with sequential late radiation therapy, early radiation therapy with poor implementation of chemotherapy did not improve the survival 11. Therefore, 3-4 cycles of induction chemotherapy before TRT for LS-SCLC with bulky tumor was often performed in clinic, which also made up time for preparing TRT.

As for the sequencing between TRT and chemotherapy after multi-cycles of induction chemotherapy for LS-SCLC with bulky tumor, there were still different viewpoints. The high incidence of radiation-related toxicity may be the main barriers in the administration of traditional concurrent chemoradiotherapy (CCRT). It was reported that two schedules of etoposide in combination with cisplatin did not result in significant differences in tumor response and survival. Moreover, the hematologic toxicity occurred more frequently in intravenous etoposide treatment schedule 12. Thus the oral formulation might provide a safer alternative of the chemotherapy regimen to perform CCRT.

So, we conducted a phase II randomized study to evaluate the efficacy and safety of concurrent TRT in combination with cisplatin and oral etoposide after third- or fourth- cycle induction chemotherapy for LS-SCLC with bulky tumor.

## Material and Methods

### Patients

**Figure 1:**
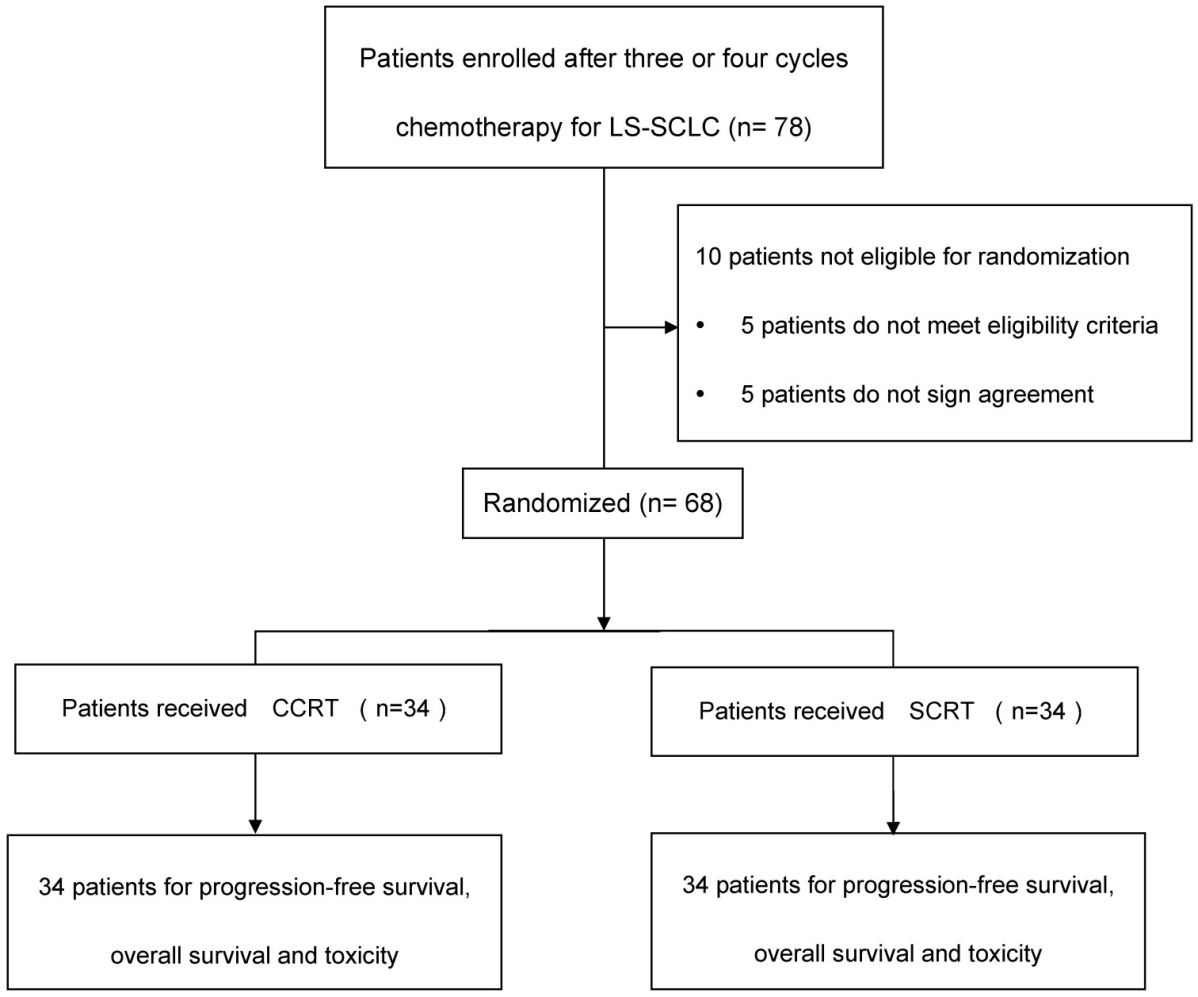
Study flow diagram. LS-SCLC, limited-stage small-cell lung cancer; CCRT, concurrent chemoradiotherapy; SCRT, sequential chemoradiotherapy.

The study flow diagram was depicted in Figure 1. Included criteria: pathologically diagnosed as SCLC, stage IIIA or IIIB disease according to the 7th edition of the American Joint Committee on Cancer (AJCC) staging system; an Eastern Cooperative Oncology Group (ECOG) performance status score of 0 or 1; aged 18 years or older; had received three or four cycles of platinum plus etoposide doublet chemotherapy. Adequate hematological, renal, hepatic and pulmonary functions were defined as: granulocytes ≥ 2.0×10^9^/L, platelets ≥100x10^9^/L, hemoglobin ≥8g/L, total bilirubin ≤1.5x upper normal limit, aspartate aminotransferase, alanine aminotransferase≤2.5× upper normal limit, creatinine ≤1.5mg/L, and FEV1 ≥1.5 L. Ability to understand and willingness to sign a written informed consent form. Exclusion criteria included: history of operation of lung cancer; progressive disease (PD) after 3-4 cycles chemotherapy; severe infection; uncontrollable diabetes; in pregnancy or lactation; currently receiving or have received other clinical trial for radioprotection within the prior six months; history of malignancy other than skin cancer or carcinoma in-situ within 2 years; history of cardiovascular diseases that might include one of the following: myocardial infraction, angina, coronary angioplasty, congestive heart failure, stroke, or coronary bypass surgery in the last 6 months; concomitant treatment with other anticancer drugs. Patients had clinical complete response (cCR) or clinical partial response (cPR) before randomization. This study was approved by the institutional review board at Tianjin Medical University Cancer Institute & hospital. All patients were required to provide written approved consent before enrollment. The study was conducted according to the protocol, Good Clinical Practice guidelines, applicable local regulations and the Declaration of Helsinki.

### Chemotherapy

Induction chemotherapy was performed in a 21-day cycle, consisting of etoposide (50 mg/m^2^ intravenously) on day 1 to 5 and cisplatin (25 mg/m^2^ intravenously, PE) on day 1 to 3 (carboplatin AUC = 5 on day 1, CE). After third or fourth cycle induction chemotherapy, chemotherapy was given in a 28-day cycle in the concurrent arm and a 21-day cycle in the sequential arm. Chemotherapy consisted of etoposide (100 mg/day orally) on day 1 to 5 and cisplatin (25 mg/m^2^ intravenously) on day 1 to 3 from the beginning of radiotherapy in the concurrent arm. Chemotherapy was the same as induction strategy in the sequential arm. If the leukocyte count was less than 3,000/mm^3^ or the platelet count lower than 75,000/mm^3^ on the first day of chemotherapy cycle, chemotherapy was withheld until the counts recovered. If patients experienced grade 4 hematologic toxicity during chemotherapy, the dose of etoposide was reduced to 75% of the initial dosage. Chemotherapy was suspended in patients with serum creatinine levels of ≥1.5× upper normal limit, serum bilirubin levels of ≥1.5× upper normal limit, or the hepatic transaminase level of ≥2.5× upper normal limit.

### Thoracic Radiotherapy

TRT started on day after the third or fourth cycle of induction chemotherapy. Intensity modulated radiation therapy (IMRT) was used. The gross tumor volume (GTV) included the primary tumor after induction chemotherapy and positive lymph nodes. The clinical tumor volume (CTV) included a 0.6 cm margin around the mass, the ipsilateral hilum and the mediastinal or supraclavicular area with positive lymph nodes. The planning target volume (PTV) was outlined around the CTV with a margin of 0.5 cm. The dose delivered by 95% of the PTV should be more than 100% of the prescription dose. Radiotherapy was performed once-daily (60Gy in 30 daily fractions of 2Gy over 42 days, on 5 consecutive days a week). The dose constraints to organs at risk (OAR) were as follows: lungs V_5_ ≤45%, V_20_ ≤30%, and the mean lung dose ≤18Gy, heart V_40_ ≤30%, esophageal V_55_ <50%, the maximum dose to the spinal cord ≤45Gy. TRT was suspended if patient experienced grade 4 hematologic toxicity, radiation pneumonitis or had difficulty in swallowing a liquid diet. The maximum spinal cord dose was limited to 45Gy.

Prophylactic cranial irradiation (PCI) (25Gy in 10 fractions over 2 weeks, on 5 consecutive days a week) was administered to responding patients who had a negative MR brain scan after completion of TRT and all chemotherapy. Parallel opposing fields were used, with 6 MV X-ray a linear accelerator. The whole brain was irradiated (with the inferior border following a line drawn to avoid the eyes), including the temporal fossae and the intracranial portion of the cranial nerves.

### Treatment evaluation and follow-up

After completion of chemoradiotherapy, the patients were followed up with physical examinations, imaging (thorax computed tomography and ultrasonography of the neck and upper abdomen) and blood tests.

The follow-up studies and the evaluation of tumor recurrence were as following. Patients were followed up every 4 weeks after the completion of therapy, every 3 months (±1 month) for 2 years, every 6 months (±1 month) for 3 years, and then annually thereafter. Recurrences were recorded when the first image showed abnormalities.

### Statistical analysis

The primary end point was progression-free survival (PFS). PFS was defined as the length of time from the date of first chemotherapy to the date of first documentation of relapse of SCLC or any other type of cancer or death. The secondary end points included OS and toxicity. OS was defined as the length of time from the date of first chemotherapy to the date of death of various reasons. Failure patterns were defined as the first site of disease progression. Local failure was defined as the persistence or recurrence of primary tumor. Regional failure was defined as the recurrence of mediastinal to supraclavicular regional lymph nodes without distant metastasis. Local-regional failure was defined as the recurrence of both local and regional. All those failures were diagnosed by follow-up CT, positron emission tomography images and ultrasonography, et al. Treatment-related toxicity was evaluated according to the National Cancer Institute Common Toxicity Criteria (version 4.0).

This study was designed as a single center, prospective, randomized phase II study. The sample size was designed to provide 80% power to detect a difference in median PFS time of 24 months versus 12 months in favor of the concurrent arm at the 0.05 error level with a two-sided test. The target sample size was 60 patients. Considering a 10% dropout rate, the total planned sample size was 66 patients. Patients were randomly assigned using minimization, with stratification by TNM stage, the cycle times of induction chemotherapy.

We calculated Kaplan-Meier curves for PFS, OS, local, regional or local-regional recurrence-free survival (LRRFS), and distant metastasis-free survival (DMFS). Differences between pairs of Kaplan-Meier curves were examined using the log-rank test. The Fisher's exact test was used to compare local, regional, and distant recurrence rates. A p-value of <0.05 was considered statistically significant. All statistical analyses were carried out using the Statistical Package for Social Sciences (SPSS) software v.21.0 (SPSS, Inc., Chicago, IL).

## Results

### Patient Characteristics

Between July 2012 and September 2015, a total of 68 patients with LS-SCLC treated in Tianjin Medical University Cancer Hospital were enrolled into the study. The last patient was enrolled in September, 2015. The median duration of follow-up was 63.3 months (95% confidence interval [CI], 50.8 to 75.8 months) using reverse Kaplan-Meier method. The characteristics of the 68 eligible patients were shown in Table 1. They were well balanced between the arms.

### Treatment Completion

All eligible patients completed 3-4 cycles of induction chemotherapy. 38.2% (13/34) of patients in the concurrent arm and 29.4% (10/34) of patients in the sequential arm received PE induction chemotherapy. The rest patients received CE induction regimen. Radiotherapy terminated in one patient in each arm because of pulmonary infection at 46Gy (concurrent arm) and radiation esophagitis at 40Gy (sequential arm), individually. The others completed the planned TRT at 60Gy. In concurrent arm, 67.6% of the patients completed full (two) cycles of concurrent chemotherapy.

### Survival

**Figure 2:**
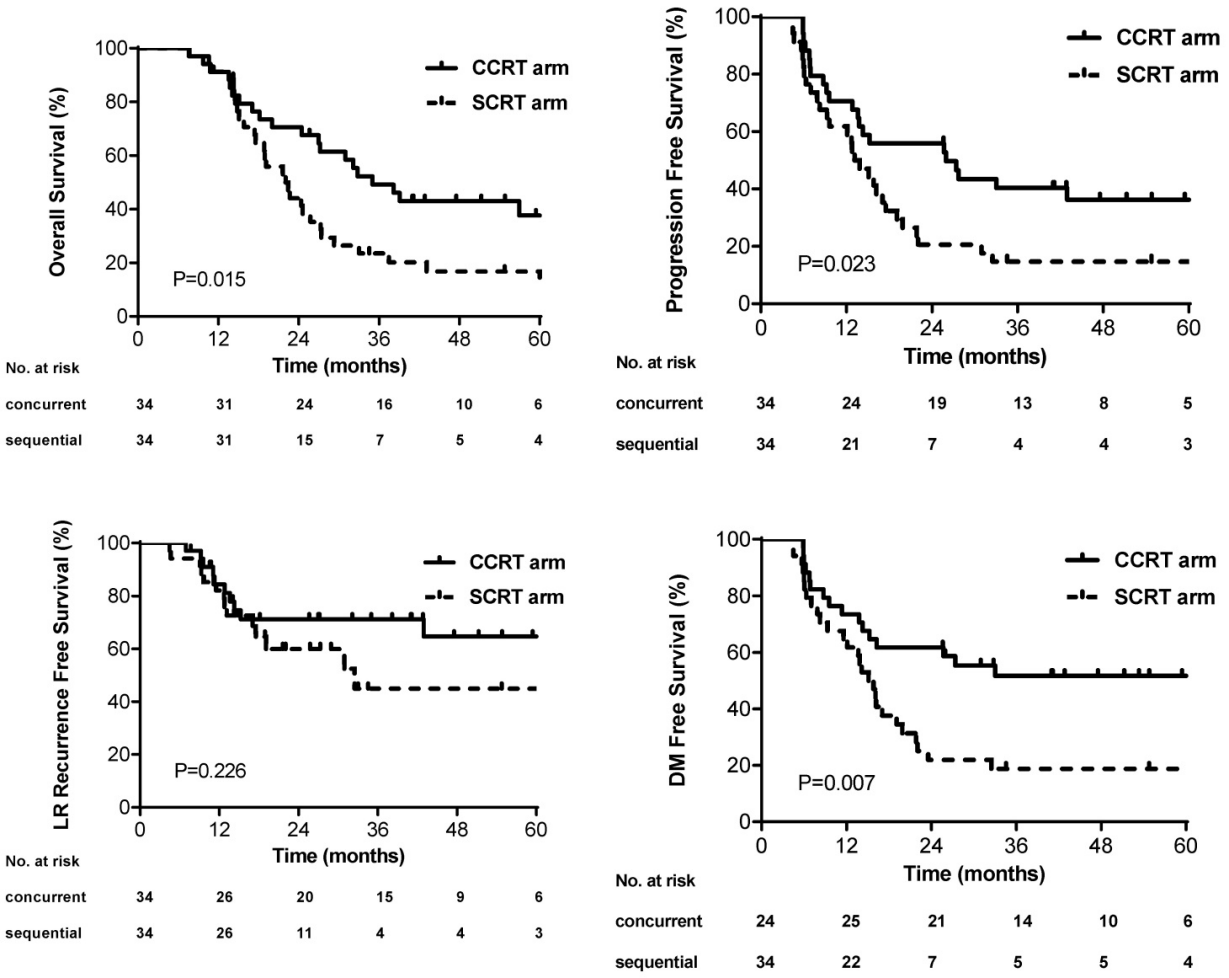
Kaplan-Meier curves for (A) overall survival, (B) progression-free survival, (C) local, regional or local-regional recurrence-free survival, and (D) distant metastasis-free survival.

The median PFS was 26.0 months (95% CI, 9.0-43.0) and 13.1 months (95% CI, 9.7-16.6) in the concurrent and sequential arm, respectively. The 3-year PFS rate was better in the concurrent arm than that in the sequential arm (40.4% versus 14.7%, p=0.023) (Figure 2A). The median OS and 3-year OS rate in the concurrent arm were 35.0 months and 49.2%, which were also improved compared with that in the sequential arm (22.0 months and 23.5%) (p=0.015) (Figure 2B). No difference of 3-year LRRFS rates was found between the two arms (45.0% sequential versus 71.2% concurrent, p=0.226) (Figure 2C). The 3-year DMFS rates was higher in the concurrent arm (51.7%) than that in the sequential arm (18.8%), which showed significant difference (p=0.007) (Figure 2D). The univariate analysis of PFS for all patients revealed that the patients who received concurrent chemoradiotherapy (HR: 0.52; 95CI%, 0.30-0.92; p=0.026) and who without cigarette smoking (HR: 0.41; 95%CI, 0.22-0.78; p=0.006) had longer PFS (Table S1). Statistical significance of these variables persisted upon multivariable analysis (Supplementary materials). Moreover, four cycles of induction chemotherapy (HR: 1.74; 95%CI, 0.96-3.15; p=0.069) tended to decrease PFS compared with three cycles of induction chemotherapy. Similar results in OS were found by the cox regression, besides that older patients appeared to have poor survival outcomes (HR: 1.68; 95%CI, 0.93-3.02, p=0.083) (Table S2).

### Patterns of Failure

A total of 20 (58.8%) and 29 (85.3%) patients experienced disease progression in the concurrent and sequential arm, respectively (p=0.015). Local, regional or local-regional failure occurred in 10 (29.4%) and 13 (38.2%) patients in the concurrent and sequential arm, individually. The distribution of the first progression at local, regional or local-regional sites was close between two arms (p=0.442). Similarly, there was no significant difference achieved in the incidence of distant metastasis as first progression site (p=0.134), although it tended to be higher in the sequential arm. Brain metastasis occurred more commonly in the sequential arm (p=0.018). The distribution of first progression was summarized in Table 2.

### Toxicity

The treatment-related toxicity was shown in Table 3. Leukopenia was the most treatment-related toxicity in both arms, but it was severer in the concurrent arm (p=0.052). Grade 3 or 4 thrombocytopenia or anemia was not observed in both arms, but grade 3 or 4 leukopenia occurred in six patients in the concurrent arm and one patient in the sequential arm. The incidences of grade 2 and above esophagitis were similar in two arms (35.3% concurrent versus 29.4% sequential, p=0.795). Grade 3 esophagitis was only found in one patient in the concurrent arm. Radiation pneumonitis was more common in the concurrent arm (20.6%) than that in the sequential arm (14.7%), but the difference was not statistically significant (p=0.525). Grade 3 pneumonitis occurred in a low frequency in either arm (5.9% each). There were no grade 4 and 5 radiation esophagitis and pneumonitis. Nausea, vomiting, fatigue and skin reaction of grade 2 and above were not common in both arms, while grade 3 or 4 toxicities were rare (only one person experienced severe nausea and vomiting in the concurrent arm).

## Discussion

In the present analysis, we explored the optimal treatment for LS-SCLC with bulky tumor. Our results demonstrated that compared to sequential TRT combined with cisplatin and intravenous etoposide, concurrent TRT combined with cisplatin and oral etoposide yielded more favorable PFS and OS with acceptable treatment-related toxicity among patients who had completed 3-4 cycles of induction chemotherapy and achieved a remission. Results from multivariate analysis also confirmed the benefit of concurrent chemoradiotherapy after induction chemotherapy in PFS and OS. Our results provided a more efficient treatment strategy for LS-SCLC with bulky tumor.

The concurrent radiotherapy in combination with chemotherapy, which has been demonstrated to bring survival benefit, was regarded as the standard treatment for LS-SCLC 1-3. However, LS-SCLC patients recruited in the present study would hardly accomplish immediate concurrent chemoradiotherapy because of bulky tumor. In order to achieve a decrease in tumor volumes for patients with large tumor burden, 3-4 cycles of induction chemotherapy were often delivered before TRT in clinic. But no data has compared the efficacy and safety between concurrent and sequential TRT combined with cisplatin and oral/ intravenous etoposide after multi-cycles induction chemotherapy for LS-SCLC with bulky tumor. Therefore, the issue concerning combined therapy for LS-SCLC with large tumor burden was explored in this analysis.

Previous studies suggested that early delivery of TRT seemed to be related to long-term survival 5, 6, 11, 13-15. But a meta-analysis indicated that only patients who completed full doses of chemotherapy could benefit from early TRT 8. One possible explanation for the advantage of administering early TRT was that it might eliminate chemotherapy-resistant tumor cells which always devoted to disseminate systemically and treatment failure 6. Nevertheless, some limitations of early TRT should be taken into consideration. On one hand, since the preparation before radiotherapy was complicated, the initiation of TRT in the first cycle chemotherapy may delay, extending treatment time. On the other hand, unlike previous studies, all patients enrolled in current analysis had large tumor burden which required large radiation target volumes. Induction chemotherapy contributed to tumor shrinkage and significant reduction of radiation planning, decreasing toxicity in normal tissue 16, 17. Considering this study included LS-SCLC patients with large tumor burden, induction chemotherapy was performed before TRT.

As regard to optimal timing of TRT related to induction chemotherapy, a Korean randomized controlled trial compared TRT starting in the first cycle of chemotherapy with TRT starting in the third cycle for LS-SCLS, and found that TRT delivered with the third cycle of chemotherapy was noninferior to early TRT in treatment compliance and clinical outcomes, but with lower toxic effects 10. Besides, multi-cycles of chemotherapy might make up time for preparing TRT. Considering the large tumor burden of the recruited patients, all enrolled were required to have completed 3-4 cycles of induction chemotherapy before received TRT. Although excessive chemotherapy might extend the overall treatment course and affect the clinical efficacy, a few patients recruited into this study ultimately experienced 4 cycles induction chemotherapy due to bulky tumors and low response to chemotherapy. The results of multivariate Cox regression analysis also revealed no statistically significant difference in PFS and OS between the cycles of induction chemotherapy (Supplementary materials). Compared with the results of late TRT arm in Korean study, the concurrent TRT after induction chemotherapy in this study resulted in an absolute superior PFS (median, 26.0 versus 11.2 months) and OS (median, 35.0 versus 26.8 months). All patients in our study achieved cCR or cPR after induction chemotherapy and then received TRT using IMRT, which may contribute to the favorable survival outcomes.

Generally, sequential rather than concurrent chemoradiotherapy was the usual choose after 3-4 cycles of induction chemotherapy 18, 19 because of the high incidence of toxicity. Additionally, intravenous etoposide and cisplatin was commonly used to combine with TRT. In our study, oral etoposide and cisplatin combined with TRT was delivered in the concurrent arm. In comparison with intravenous etoposide, oral administration of etoposide resulted in relatively lower toxicity but no inferior survival outcome 12, 20-22. Furthermore, the oral formulation provides a more convenient alternative for patients, and exhibits advantages of cost saving for hospital stays comparing with the intravenous one. Thus, oral etoposide and cisplatin were adopted as the chemotherapy regimen in concurrent arm in our study. Compared with previous studies 8, 13, 14, 23, the incidence of radiation esophagitis in this analysis was not higher, partly owing to the oral administration of etoposide in concurrent arm. The incidence of grade 2 radiation pneumonitis was relatively higher with partial explanation of more radiation exposure of normal lung tissue in LS-SCLC patients who had bulky tumors and needed larger radiotherapy volume. But sever radiation pneumonitis was rare.

As regards radiation dose and fraction, we adopted once-daily TRT in 2.0Gy over 6 weeks to a total dose of 60Gy in current analysis. The optimal radiotherapy schedule and dose was explored in the landmark Intergroup 0096 study, which demonstrated significant improvement in survival rates with twice-daily concurrent chemotherapy (45Gy/30 fractions over 3 weeks) compared to once-daily treatment (45Gy/25 fractions over 5 weeks) 24. However, the higher rate of severe esophagitis in the twice-daily arm, the lower biologically effective dose of radiation in once-daily arm, and the predominately 2-dimensional treatment planning of that area resulted in its limitations in clinical adoption. But then, the CONVERT trial showed no significant difference in survival and toxicity between twice-daily (45Gy in 30 twice-daily fractions of 1.5Gy over 19 days) and once-daily (66Gy in 33 daily fractions of 2Gy over 45 days) concurrent chemoradiotherapy in LS-SCLC patients, using modern conformal radiotherapy techniques 23. In fact, considering the convenience for patients and logistical ease 25, we preferred once-daily therapy in this study.

This study is limited by a relatively small number of enrolled patients, which might limit the generalizability of our conclusions. The survival benefits and safety of concurrent chemoradiotherapy with oral etoposide and cisplatin should be further explored in larger scale randomized controlled trials.

In conclusion, concurrent thoracic radiotherapy in combination with cisplatin and oral etoposide, after completing 3-4 cycles of induction chemotherapy and achieving a remission, significantly improved PFS and OS with acceptable treatment-related toxicity. Hence this is a promising therapeutic strategy for limited-stage small-cell lung cancer with bulky disease.

## Supplementary Material

Supplementary figures and tables.

## Figures and Tables

**Table 1:** Patient and disease characteristics at baseline

Characteristics	Concurrent arm (n=34)		Sequential arm (n=34)		P-value
	n	%	n	%	
**Gender**					0.417
Male	26	76.5	23	67.6	
Female	8	23.5	11	32.4	
**Age, years**					0.801
≤60	22	64.7	21	61.8	
>60	12	35.3	13	38.2	
Median	57		57		0.102
Range	29-71		48-73		
**Weight loss**					0.487
Yes	5	14.7	7	20.6	
No	29	85.3	27	79.4	
**Cigarette smoking**					0.798
Yes	23	67.6	22	64.7	
No	11	32.4	12	35.3	
**Alcohol consumption**					0.793
Yes	10	29.4	11	32.4	
No	24	70.6	23	67.6	
**Tumor location**					
Left lung	19	55.9	10	29.4	0.051
Right lung	12	35.3	22	64.7	
Mediastinal SCLC	3	8.8	2	5.9	
**T stage**					0.429
T1	3	8.8	3	8.8	
T2	8	23.5	15	44.1	
T3	8	23.5	7	20.6	
T4	14	41.2	8	23.5	
Tx	1	2.9	1	2.9	
**N stage**					0.418
N1	1	2.9	1	2.9	
N2	25	73.5	20	58.8	
N3	8	23.5	13	38.2	
**AJCC clinical stage**					0.625
IIIA	20	58.8	18	52.9	
IIIB	14	41.2	16	47.1	
**Histological type**					0.100
SCLC	31	91.2	26	76.5	
Combined SCLC	3	8.8	8	23.5	
**Induction chemotherapy cycles**					0.793
3	24	70.6	23	67.6	
4	10	29.4	11	32.4	
**Induction chemotherapy regimen**					0.442
PE	13	38.2	10	29.4	
CE	21	61.8	24	70.6	

AJCC, American Joint Committee on Cancer; CE, carboplatin and etoposide; PE, cisplatin and etoposide.

**Table 2:** First progression site according to treatment arms

Site	Total arm (n = 68)		Concurrent arm (n = 34)		Sequential arm (n = 34)		P-value
	n	%	n	%	n	%	
No recurrence	19	27.9	14	41.2	5	14.7	0.015
Local	6	8.8	3	8.8	3	8.8	1.000
Regional	4	5.9	3	8.8	1	2.9	0.303
Distant metastasis	26	38.2	10	29.4	16	47.1	0.134
Local-regional	3	4.4	1	2.9	2	5.9	0.555
Local-metastasis	4	5.9	2	5.9	2	5.9	1.000
Regional-metastasis	4	5.9	1	2.9	3	8.8	0.303
Local-regional and metastasis	2	2.9	0	0	2	5.9	0.151
Local, regional or local-regional	23	33.8	10	29.4	13	38.2	0.442
Any site	49	72.1	20	58.8	29	85.3	0.015

**Table 3:** Treatment toxicity according to treatment arms

Toxic effect	Concurrent arm (n = 34)						Sequential arm (n = 34)				P-value
	Grade 2-4	Grade 2	Grade 3		Grade4		Grade 2-4	Grade 2	Grade 3	Grade4	
Leukopenia	20(58.9)	14(41.2)		4(11.8)		2(5.9)	12(35.3)	11(32.4)	0(0.0)	1(2.9)	0.052
Thrombo-cytopenia	1(2.9)	1(2.9)		0(0.0)		0(0.0)	1(2.9)	1(2.9)	0(0.0)	0(0.0)	1.000
Anemia	1(2.9)	1(2.9)		0(0.0)		0(0.0)	0(0)	0(0.0)	0(0.0)	0(0.0)	1.000
Radiation esophagitis	12(35.3)	11(32.4)		1(2.9)		0(0.0)	10(29.4)	10(29.4)	0(0.0)	0(0.0)	0.795
Radiation pneumonia	7(20.6)	5(14.7)		2(5.9)		0(0.0)	5(14.7)	3(8.8)	2(5.9)	0(0.0)	0.525
Nausea	6(17.6)	5(14.7)		1(2.9)		0(0.0)	0(0.0)	0(0.0)	0(0.0)	0(0.0)	0.033
Vomiting	3(8.8)	2(5.9)		1(2.9)		0(0.0)	0(0.0)	0(0.0)	0(0.0)	0(0.0)	0.238
Fatigue	4(11.8)	4(11.8)		0(0.0)		0(0.0)	7(20.6)	7(20.6)	0(0.0)	0(0.0)	0.510
Skin reaction	3(8.8)	3(8.8)		0(0.0)		0(0.0)	2(5.9)	2(5.9)	0(0.0)	0(0.0)	1.000
											
