# Supplementary material for: Concurrent or Sequential Chemoradiotherapy after 3-4 Cycles Induction Chemotherapy for LS-SCLC with Bulky Tumor
Source: J Cancer. 2020 Jun 16;11(17):4957–64. doi: 10.7150/jca.41136 (PMC7378916; doi:10.7150/jca.41136)
Supplement: Supplementary file 1 — Supplementary figures and tables. [file jcav11p4957s1.pdf]

Table S1 Exploratory Cox regression analysis of progression-free survival predictors

Variables	Univariate analysis		Multivariate analysis	
	HR (95% CI)	P-value	HR (95% CI)	P-value
Gender				
Male	1.00		-	-
Female	0.62 (0.33-1.20)	0.155	-	-
Age, years				
≤60	1.00		-	-
>60	1.48 (0.84-2.61)	0.177	-	-
Weight loss				
Yes	1.00		-	-
No	0.65 (0.33-1.31)	0.228	-	-
Cigarette smoking				
Yes	1.00		1.00	
No	0.41 (0.22-0.78)	0.006	0.41 (0.20-0.83)	0.013
Alcohol consumption				
Yes	1.00		-	-
No	1.00 (0.54-1.82)	0.987	-	-
AJCC clinical stage				
IIIA	1.00		1.00	
IIIB	1.64 (0.94-2.86)	0.084	1.09 (0.59-2.02)	0.775

Induction chemotherapy cycles				
3			1.00	
4	1.74 (0.97-3.14)	0.065	1.74 (0.96-3.15)	0.069
Concurrent chemotherapy				
No	1.00		1.00	
Yes	0.52 (0.30-0.92)	0.026	0.48 (0.27-0.86)	0.013

AJCC, American Joint Committee on Cancer; CI: confidence interval; HR, hazard ratio

Table S2 Exploratory Cox regression analysis of overall survival predictors

Variables	Univariate analysis		Multivariate analysis	
	HR (95% CI)	P-value	HR (95% CI)	P-value
Gender				
Male	1.00		-	-
Female	0.60 (0.31-1.17)	0.132	-	-
Age, years				
≤60	1.00		1.00	
>60	1.63 (0.92-2.91)	0.097	1.68 (0.93-3.02)	0.083
Weight loss				
Yes	1.00		-	-
No	0.82 (0.41-1.66)	0.586	-	-

Cigarette smoking				
Yes	1.00		1.00	
No	0.44 (0.23-0.84)	0.013	0.45 (0.23-0.88)	0.019
Alcohol consumption				
Yes	1.00		-	-
No	1.11 (0.60-2.03)	0.745	-	-
AJCC clinical stage				
IIIA	1.00		-	-
IIIB	1.50 (0.86-2.64)	0.157	-	-
Induction chemotherapy cycles				
3			1.00	
4	1.70 (0.94-3.08)	0.077	1.60 (0.88-2.91)	0.122
Concurrent chemotherapy				
No	1.00		1.00	
Yes	0.50 (0.28-0.88)	0.017	0.45 (0.25-0.82)	0.008

AJCC, American Joint Committee on Cancer; CI: confidence interval; HR, hazard ratio
